# Gene therapy for neovascular age-related macular degeneration: rationale, clinical trials and future directions

**DOI:** 10.1136/bjophthalmol-2020-316195

**Published:** 2020-04-08

**Authors:** Thales Antonio Cabral de Guimaraes, Michalis Georgiou, James W B Bainbridge, Michel Michaelides

**Affiliations:** 1 UCL Institute of Ophthalmology, University College London, London, UK; 2 Moorfields Eye Hospital, London, UK

**Keywords:** angiogenesis, clinical trial, degeneration, genetics, retina

## Abstract

Age-related macular degeneration (AMD) is one of the leading causes of irreversible blindness in the developed world. Antivascular endothelial growth factor therapy has transformed the management and outcome of neovascular AMD (nAMD), although the need for repeated intravitreal injections—even lifelong—and the related complications, high drug costs, frequent clinic visits and repeated imaging have resulted in an enormous burden both to healthcare systems and patients. The application of gene therapy approaches for sustained delivery of a range of antiangiogenic proteins has the promise of helping to address these aforementioned challenges. A number of early phase clinical trials of gene therapy in nAMD have provided encouraging results, with many more ongoing or anticipated. There remain significant areas of controversy, including regarding the optimal treatment targets, routes of administration and potential safety concerns. In this review we aim to provide an update of the current status of gene therapy for nAMD and briefly discuss future prospects.

## Introduction

Age-related macular degeneration (AMD) is a progressive disease that leads to severe impairment of central vision. It is a leading cause of irreversible blindness in the elderly population.^1–3^ A large systematic review and meta-analysis predicted the number of patients affected by the condition worldwide to be 288 million by 2040.^4^ In Europe alone, late AMD is expected to affect 77 million people by 2050.^5^

AMD can be categorised into two main groups: dry or non-neovascular AMD, and wet or neovascular AMD. Neovascular AMD (nAMD) presents with the growth of abnormal vessels, most often emanating from the choroidal circulation, but can originate from the retinal vasculature, causing acute vision loss through leakage, and if chronic through fibrosis and atrophy.^6^ While a complex array of proangiogenic and antiangiogenic factors are involved in vascular homeostasis, it appears that nAMD is primarily driven by a perturbation of vascular endothelial growth factor (VEGF), demonstrated by the dramatic improvements observed in patients with nAMD following the development of anti-VEGF agents. This class of drug is now the gold standard treatment for nAMD.^7^ These intravitreal drugs inhibit neovascular proliferation by directly binding to VEGF or to VEGF receptors (VEGFR). However, the high drug costs and the frequent need for intravitreal injections have resulted in a significant (arguably unsustainable) burden, economically, logistically and socially.^8^ Long-term follow-up studies have shown that visual acuity (VA) gains during the first years of treatment were not maintained and that there was a progressive decline of VA gains achieved with repeated monthly treatment.^9–11^ Moreover, a recent meta-analysis suggested that treatment intensity correlated directly with final visual outcomes (with real-world experience often being of ‘under-treatment’ and thereby inferior outcomes compared with those achieved in pivotal clinical trials), further highlighting the need for a more sustainable release approach.^12^

Gene therapy offers the very real possibility of such a sustained delivery—in a ‘one and done’ manner. The gene therapy for *RPE65*-associated retinal dystrophy approved by the Food and Drug Administration and the European Medicines Agency has helped pave the way for a new era of retinal gene therapy products.^13–15^ Gene therapy for nAMD is on the verge of transforming management and outcomes, as intravitreal anti-VEGF agents did a decade ago. This review aims to provide an update on targets, trials and future directions.

## Molecular basis of angiogenesis in nAMD

Angiogenesis is a complex and critical physiological process which is tightly regulated by proangiogenic and antiangiogenic factors.^16^ In ocular disease, in the presence of hypoxia, retinal neurons such as the retinal ganglion cells release growth factors which upregulate the secretion of VEGF by Muller cells and retinal microglia.^17^ Although several factors are involved in this physiological process, including placental growth factor (PIGF), platelet-derived growth factor (PDGF-β), angiopoietin-1 (Ang1) and angiopoietin-2 (Ang2), VEGF plays the major role, primarily regulating vascular permeability and neovascularisation.^16 18–22^ It has multiple isoforms due to alternative splicing, although VEGF_165_ is the predominant isoform and most active in vasculogenesis.^23–25^

**Figure 1:**
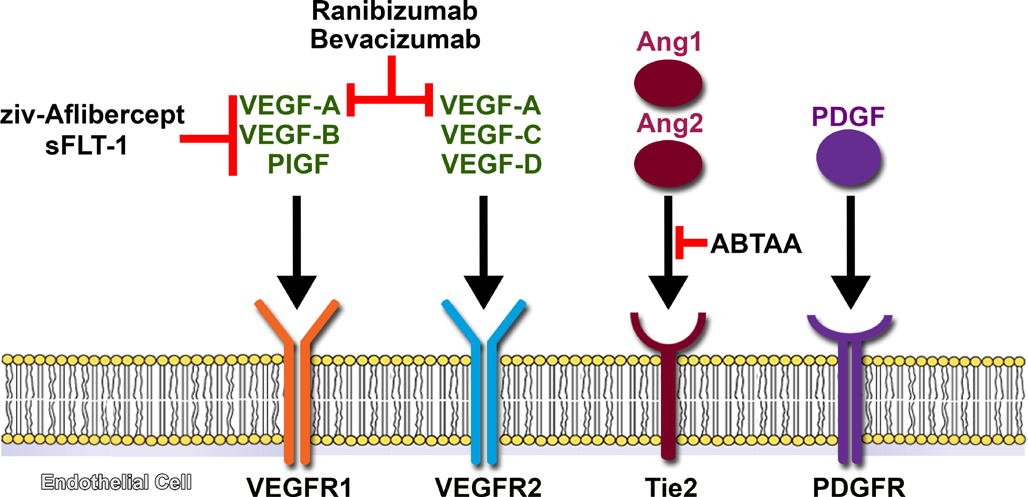
Promising targets for antiangiogenesis in nAMD. The main goal of gene therapy in this condition is to continuously express antiangiogenic factors and lead to a more sustainable treatment. Some have already been extensively studied in the past for intravitreal treatment of nAMD, like bevacizumab and ranibizumab, while others like Angpt2-binding and Tie2-activating antibodies (ABTAA) are currently being investigated and might be useful for combination therapy. The platelet-derived growth factor (PDGF) pathway also represents another potential target as it mediates the recruitment and survival capabilities of pericytes and might be implicated in the development of subretinal fibrosis. Ang1, angiopoietin-1; Ang2, angiopoietin-2; nAMD, neovascular age-related macular degeneration; PIGF, placental growth factor; VEGF, vascular endothelial growth factor.

Among VEGF’s signalling receptors, the endothelial and neuron-expressed VEGFR1 (*FLT1*) and VEGFR2 (*FLK1/KDR*) are responsible for regulation of cell proliferation, migration and vascular permeability in ocular disease, whereas VEGFR3 is mainly expressed in lymphatic endothelial cells and is mostly responsible for lymphangiogenesis.^20 26^ VEGF-A binds with high affinity to both VEGFR1 and VEGFR2, while VEGF-B and PIGF bind exclusively to *VEGFR1* (figure 1).^27–29^ Therefore, the vast majority of gene therapy trials for nAMD target VEGF, either directly or indirectly via its receptors, although other factors are also being investigated as possible targets and will be reviewed in the following sections.

## Viral vectors and the eye

The eye is arguably the ideal target for gene therapy: it has relative immune privilege, since it is separated from the rest of the body by the presence of the inner blood–retinal barrier; it is highly compartmentalised, which allows easy access to different and specific ocular tissues; the transparency of the ocular media allows the direct visualisation of the retina; and visual function and retinal architecture can be monitored readily using non-invasive methods.^30^ A prerequisite for gene therapy to be successful is a vector that leads to sustained levels of gene expression, low toxicity and immunogenicity. Various non-viral and viral vector systems have been used in different settings, but the most suitable for retinal gene supplementation is the recombinant adeno-associated viral vector (AAV).^31 32^

A major reason for the success of AAV is its small, single-stranded DNA genome of about 4.6 kilobases (kb) and capsid organisation, which facilitate genetic modification, thus enabling customisation of its properties.^33^ However, given the fact that approximately 6% of all human proteins have a coding sequence bigger than 4 kb, this small packaging capability is also a limitation of the first-generation AAV2.^34 35^ Several strategies have been investigated to bypass this cargo capacity, including directed evolution and the generation of dual AAV vectors. The latter aims to reconstitute a larger gene by either trans-splicing, homologous overlapping sequences or a combination of both.^36^ Other AAV serotypes have also been identified, but the most extensively studied in ocular gene therapies are AAV2, AAV5 and AAV8.^37–39^

One of the principal considerations of gene therapy is the potential immune response to AAV capsid, which may have a damaging tissue effect, but might also mitigate therapeutic benefit. AAV vector administration in humans, unlike most animal models, results in antigen-specific T-cell activation.^40^ The risk is higher during the early postoperative period. A broad range of immunosuppressive agents (both steroids and steroid-sparing agents), routes of administration (topical, periocular, intravitreal and oral) and duration (days to weeks to months) have been employed in ocular gene therapy trials, with the rationale that a relatively short course of immunosuppression in the perioperative period will ameliorate immune responses until the capsid antigens are cleared from the infected cells.^40^ As would be anticipated, studies have also demonstrated that there is a dose–response relationship between humoral immunity to AAV capsid and dose of injected AAV particles.^41^ However, this relationship ultimately limits the maximum viral particle dose that can be delivered, and may in certain situations inherently limit therapeutic benefit, thereby additionally making optimal management (ideally prevention) of immune responses critical. The route of vector delivery, as discussed in the following section, also has a major direct impact on immunogenicity, with evidence to date indicating that subretinal delivery is associated with less inflammation than the intravitreal route.^42^

An additional consideration, in direct contrast to the majority of subjects with inherited retinal diseases (IRDs) who may be targeted with gene therapy, patients with nAMD will be significantly older. This may be advantageous in terms of the likely less rigorous immune responses anticipated in older people compared with children/young adults, but also potentially disadvantageous, as older patients may be more susceptible to possible deleterious effects of systemic immunosuppression and also have pre-existing medical conditions, making them ineligible to participate and/or more likely to have side effects.

## Routes of vector delivery

**Figure 2:**
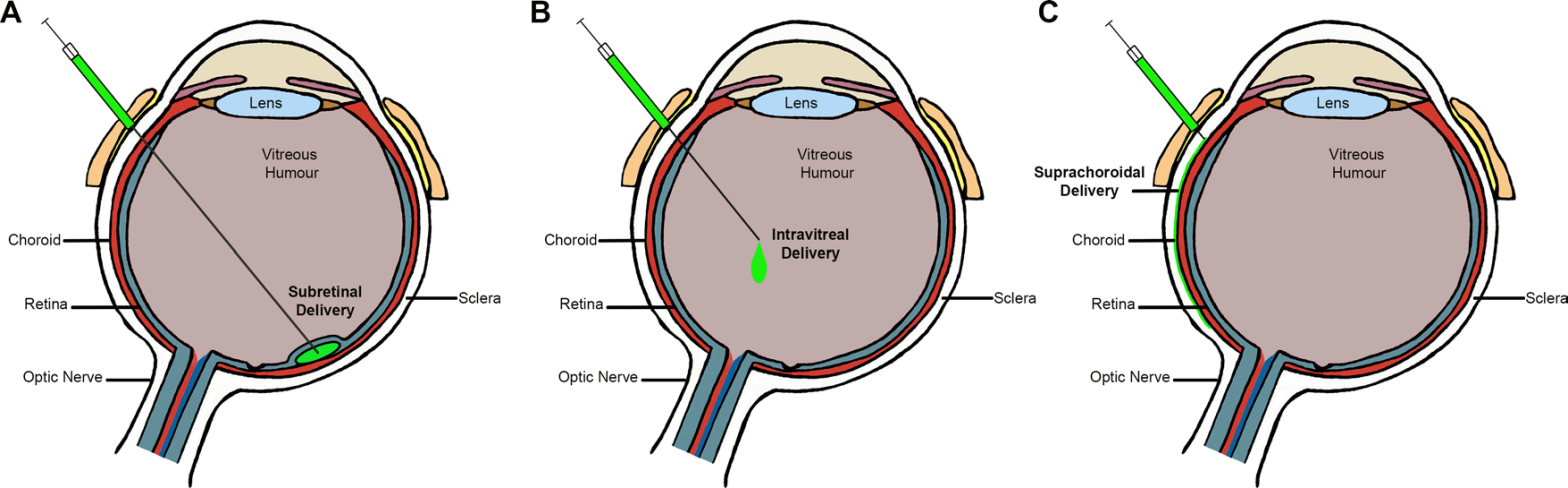
Delivery routes for gene therapy. Schematic figure of the eye demonstrating the possible routes of delivery to introduce viral vectors into the eye.

The route of administration is another major determinant of the efficacy of gene delivery. It allows the targeting of specific cell types by delivering the vector as close as possible to the desired tissue.^43^ Figure 2 offers a schematic view of the three principal modes of delivery: subretinal, intravitreal and suprachoroidal.

Although more complex and invasive than intravitreal injections, subretinal delivery is arguably preferable for diseases primarily affecting the retinal pigment epithelium (RPE) and/or photoreceptors. Since most IRDs affect either of these two cell types or both, subretinal delivery is the most commonly used route of administration in gene therapy trials for most monogenic conditions. However, intravitreal delivery has been undertaken for (1) the two X linked retinoschisis (XLRS) gene therapy trials (NCT02317887 and NCT02416622), with one stopping due to lack of efficacy and inflammation concerns (NCT02416622); and also (2) delivery of antisense oligonucleotide therapies for *CEP290* and *USH2A* variants (NCT03140969 and NCT03780257, respectively), although these do not require a viral vector. While subretinal delivery with its associated transient retinal detachment is likely to be inherently associated with greater tissue damage/disturbance compared with intravitreal, the available trial data suggest that it is well tolerated, safe and may provide effective treatment.^13 15 44^ The site of subretinal injection is also an important consideration. A retinotomy being near the (often initial retinotomy being superior) temporal vascular arcades is desirable as it allows the bleb to spread more gently towards the fovea and create a shallow elevation of the foveal region, thereby also minimising foveal stretch and reducing the deleterious effects of a localised central foveal detachment.^45–47^

Intravitreal injection of anti-VEGF agents has become the mainstay of treatment for nAMD, diabetic eye disease and other retinovascular diseases. Although much less invasive than subretinal injections and readily delivered by non-specialist surgeons, the majority of currently available AAVs are unable to reach the outer retina, including the RPE and choroid, mainly due to the inner limiting membrane (ILM) acting as a physical barrier, thereby primarily limiting transduction to the inner retinal layers. However, modified AAVs (especially serotypes 2, 8 and 9, with or without modified capsid proteins) are proposed to be more effective in penetrating the ILM and able to result in broader transduction.^48–52^ Primate data using intravitreal delivery of AAV2 have shown an annulus of high transduction in the perifoveal zone (‘ring-of-fire’).^50^ This result differs from the more uniform transduction reported after intravitreal injection in other species, including rodents.^53 54^ Additionally and equally important are immunological considerations, with intravitreal AAV administration in animal models shown to generate a humoral response against adenoviral capsid blocking further vector expression in the second eye, which did not happen with subretinal administration.^42^ Moreover, the aforementioned XLRS human clinical trial (NCT02416622) also demonstrated that the intravitreal route is more proinflammatory compared with subretinal delivery. In addition to being a significant safety concern, such immune response will likely mitigate any therapeutic effect.

Suprachoroidal delivery has also been explored and is arguably an attractive route for nAMD and other conditions affecting the choroid. Preclinical and animal data suggest that administration via suprachoroidal injection results in more posterior and circumferential distribution of the agent.^55^ Widespread expression in the RPE and photoreceptor layers has been reported 2 weeks after suprachoroidal injection using a suprachoroidal space (SCS) microinjector of AAV8 expressing an anti-VEGF Fab (AAV8.GFP) in non-human primates, pigs and rats.^56^ In this preclinical study, delivery of RGX-314 into the SCS resulted in similar suppression of VEGF-induced vascular leakage and expression of anti-VEGF Fab as subretinal delivery. Previously, access to this virtual space, between the choroid and the sclera, has been investigated by different methods, including sclerotomy. In 2011, Peden *et al* 57 described a novel technique in animal models, consisting of an ab externo approach using an illuminated microcatheter (originally designed for use in Schlemm’s canaloplasty) to inject the vector into the SCS.^58 59^ Subsequently, researchers used the iTrack microcatheter (iTRACK 400; iScience Interventional Corporation, Menlo Park, California, USA) to deliver a combination of bevacizumab and triamcinolone to the submacular SCS of 21 human subjects with nAMD.^60^ These patients were followed for a 6-month period. Complications were mild, with one eye experiencing a transient intraocular pressure elevation at 3 months postprocedure and two eyes had an apparent increase in pre-existing nuclear sclerotic cataract.^60^ The results of a phase II study to evaluate suprachoroidal injection of triamcinolone acetonide suspension in patients with macular oedema due to non-infectious uveitis (NCT02255032) were recently published with good tolerability and significantly improved VA.^61^

## Gene therapies for nAMD

Gene therapy holds the potential to continuously express therapeutic levels of angiostatic proteins. The completed and ongoing gene therapy trials for nAMD are summarised in table 1 and discussed in detail in the following sections.

**Table 1:** Completed and ongoing gene therapy clinical trials for nAMD

Trial registration number	Expressed gene	Vector	Phase	Route of delivery	Status	Sponsor	Location	Patients (n)
NCT00109499	PEDF	AAV5	I	Intravitreal	Completed	GenVec	USA	28
NCT01494805	sFLT01	AAV2	I/II	Subretinal	Completed	Lions Eye Institute, Adverum Biotechnologies	Australia	40
NCT03748784	Aflibercept	AAV2	I	Intravitreal	Ongoing	Adverum Biotechnologies	USA	30
NCT01024998	sFLT01	AAV2	I	Intravitreal	Completed	Sanofi Genzyme	USA	19
NCT03066258	Anti-VEGF Fab	AAV8	I/IIa	Subretinal	Ongoing	Regenxbio	USA	42
NCT01301443	Endostatin and angiostatin	EIAV	I	Subretinal	Completed	Oxford BioMedica	USA	21
NCT03585556	sCD59	AAV2	I	Intravitreal	Ongoing	Hemera Biosciences	USA	25

AAV, adeno-associated viral vector; EIAV, equine infectious anaemia lentiviral vector; nAMD, neovascular age-related macular degeneration.

### Pigment epithelium derived factor

The intravitreal administration of a transgene vector of AAV5 carrying pigment epithelium derived factor (*PEDF*) (AdGVPEDF.11D) in three murine models of choroidal neovascularization (CNV) significantly inhibited neovascularisation.^62^ This study led to one of the first gene therapy trials for nAMD (NCT00109499), a phase I, dose-escalation intravitreal delivery of AdPEDF.11. Although 25% of the cohort showed mild signs of inflammation, such as keratic precipitates and flare in the anterior chamber, this was readily managed, and the authors deemed this gene therapy product to be safe and well tolerated.^63^ No conclusions regarding efficacy were made as the trial did not have randomised control groups. However, patients receiving 10^8^ particle units (PU) or more of AdPEDF.11 were significantly more likely to remain stable or have a reduction in the size of CNV lesions than patients who received less than 10^8^ PU, suggesting a dose-related response.

### sFLT-1

sFLT-1 is a highly potent endogenous VEGF-A inhibitor.^64^ The Lions Eye Institute, in collaboration with Adverum Biotechnologies, completed a phase I/II randomised controlled trial involving a single subretinal administration of rAAV.sFLT-1, also known as AVA-101 (NCT01494805). All patients (n=8) received additional ranibizumab injections at baseline, week 4 and as needed afterwards according to prespecified rescue criteria. AVA-101 was shown to be safe, thereby leading to an expansion phase,^65 66^ with 32 patients randomised to either the 10^11^ vg cohort or the control group. There were no serious adverse events and patients in the therapy cohort required a median of two ranibizumab injections compared with four for the control group. Although no significant improvement in best corrected VA was seen, safety and tolerability were demonstrated, supporting the feasibility of further ocular gene therapies as a potential long-term treatment option for nAMD.

Another phase I, dose-escalation study, sponsored by Sanofi Genzyme, evaluated the safety profile of a single intravitreal AAV2-sFLT-1 injection in patients with advanced nAMD (NCT01024998). It is similar to AVA-101, except that it is a fusion protein of the sFLT-1 domain 2 with the Fc domain of IgG1. Nineteen patients were divided into four dose cohorts and followed up for 52 weeks. Four subjects showed substantial and sustained macular fluid reduction through week 52, with reductions of central subfield thickness (CST) ranging from 128 µm to 443 µm. Improvements in VA may have been limited due to pre-existing subretinal fibrosis/scarring and variability in baseline fluid. Overall the intravitreal injection was well tolerated at all doses, although one patient had transient intraocular inflammation at the highest dose (2×10^10^ vg). Another two patients had retinal haemorrhage, one had multiple retinal tears 1 month after injection, and there was a death in the study, with none of these believed to be associated with the procedure or the gene therapy product.^67^ Additionally, five out of ten subjects who received the high dose had no detectable anti-AAV2 serum antibodies. One of these patients showed no detectable sFLT-1 in the aqueous humour despite undetectable anti-AAV2 antibodies, indicating that anti-AAV2 titre and vector dose are not the only factors influencing transgene expression.^67^

### Aflibercept

Preclinical evaluation of intravitreal ADVM-022 in non-human primates was well tolerated and effective at preventing clinically relevant laser-induced CNV lesions.^68^ Adverum Biotechnologies has started a phase I, dose-escalation trial (NCT03748784) to study the safety profile of intravitreal ADVM-022 (AAV2.7m8-aflibercept), a novel recombinant AAV. Preliminary results of this human trial reveal that through week 34, in all six patients of the first cohort, the gene therapy product was well tolerated with no serious adverse events noted. Nineteen adverse events related to inflammation were noted, with the vast majority considered mild and treated with topical steroid drops only. No retinitis, vasculitis or choroiditis was seen. In addition, the improvement in CST was sustained after 34 weeks and no rescue injections were needed.^69 70^ The last update was to a median follow-up of 44 weeks, no rescue injections were needed, and anatomical and vision improvements were sustained, although one patient had spontaneous pseudophakic retinal detachment, deemed to be unrelated to the Advanced Therapy Investigational Medicinal Product.^71^ The study is still ongoing and has an estimated completion date of February 2022.

### Ranibizumab-like

RGX-314 is a recombinant AAV8 vector carrying a coding sequence for a soluble anti-VEGF protein related to ranibizumab. Regenxbio is conducting a phase I/II, dose-escalation study to evaluate the safety and tolerability of subretinal RGX-314 in subjects who have been previously treated with any anti-VEGF drug (NCT03066258). Forty-two patients were recruited and divided into five cohorts of 3×10^9^ vg, 1×10^10^ vg, 6×10^10^ vg, 1.6×10^11^ and 2.5×10^11^ vg. The trial is currently ongoing, with preliminary results being promising. In cohort 5, 73% of subjects (8 of 11) remained free of needing any anti-VEGF injections for 6 months, associated with improvement in vision and retinal thickness.^72^ After 1.5 years of RGX-314 administration, 50% of cohort 3 (3 of 6) remain free of anti-VEGF drugs and have maintained the improvement in vision. Furthermore, RGX-314 was well tolerated at all doses and no inflammation beyond what is expected following routine vitrectomy was seen.^72–74^ Regenxbio is now planning to start suprachoroidal delivery trials for nAMD and diabetic retinopathy in 2020.^72^

### Angiostatin and endostatin

RetinoStat is an equine infectious anaemia lentiviral vector that expresses angiostatin and endostatin, both of which are inhibitors of angiogenesis. Animal models of rhesus macaques and rabbits have shown that subretinal administration was safe and capable of persistent expression.^75^ In a phase I, dose-escalation trial, 21 patients were enrolled into three cohorts. Although one patient developed a procedure-related macular hole, the subretinal injection of RetinoStat was safe and well tolerated, resulting in sustained transgene expression of VEGF-neutralising proteins (NCT01301443), established via analysis of aqueous samples.^76^ A concomitant long-term follow-up study is currently under way (NCT01678872).

### Complement cascade

Hemera Biosciences recently started two phase I trials using intravitreal AAVCAGsCD59 for both nAMD and dry AMD (NCT03585556 and NCT03144999, respectively). This molecule targets the terminal step of complement activation that leads to the formation of the membrane attack complex.^77^ Interestingly, it has been shown that subretinal injection of AAV-CD59 attenuated the formation of laser-induced CNV by around 60% in mice, even when the site of delivery was distal to the laser-induced CNV site.^78^

## Other possible targets and approaches for gene therapy

Gene therapies for nAMD are largely focused on the use of vector systems to express antiangiogenic proteins that either directly or indirectly block the VEGF pathway as discussed earlier. Other possible targets for gene therapy are discussed in the following sections and presented in figure 1.

### Platelet-derived growth factor

*PDGF* mediates the recruitment and survival capabilities of pericytes. *PDGF* has been implicated in the pathogenesis of several fibrotic conditions and may also play a role in the development of subretinal fibrosis.^79 80^

### Tie-2 tyrosine kinase receptor

Tie-2 tyrosine kinase receptor is a potential target present in endothelial cells. The angiopoietin (Angpt)–Tie2 system functions as a key regulator of adult vascular homeostasis.^81^ In animal models, intravitreal administration of Ang1 was as effective as VEGF-trap in inhibiting CNV formation. Furthermore, Ang1 suppressed vascular leakage by increasing endothelial junctional proteins.^82^ More recently, an intravitreal dual functioning antibody, Angpt2-binding and Tie2-activating (figure 1), has been shown to suppress CNV formation and vascular leakage, and also improve hypoxia surrounding the CNV, thereby making it an attractive option for combination therapy with anti-VEGF drugs.^83^

### Gene silencing

Gene silencing has been attempted via small RNA interference (siRNA), which has been shown to successfully suppress expression of VEGF in mice models.^84^ However, a phase III human trial administering the siRNA bevasiranib intravitreally (NCT00499590) was discontinued as recommended by the study’s Independent Data Monitoring Committee due to the unlikelihood of reaching its primary objective, defined as the proportion of patients at week 60 in each group with a successful VA outcome.^85^ Similarly, a phase II trial administering Sirna-027 (NCT00395057) failed to meet key efficacy endpoints, despite positive reports of the phase I/II study (NCT00363714).^86^

In a study by Askou *et al* 87, AAV2/8-mediated subretinal delivery of a short hairpin RNA targeting *VEGF* has been found to be efficient and to reduce formation of CNV in preclinical murine models of wet AMD. An alternative to siRNAs is the use of microRNAs, a method that has been previously investigated by the same group of researchers. Intramuscular delivery of AAV containing polycistronic miRNA clusters efficiently silenced *VEGF* as early as 21 days after delivery in mice.^88^

## Conclusions and future directions

In summary, AMD is a complex disease with a multitude of pathways involved in its pathogenesis, with this heterogeneity posing therapeutic challenges. The approach to date has been to continuously express anticomplement and/or antiangiogenic proteins (especially targeting VEGF). There is no doubt that gene therapy holds promise to ameliorate the social and economic burdens associated with chronic anti-VEGF therapy by providing reliable and sustainable release of antiangiogenic drugs.

Several early phase trials have shown significant promise, with final results expected from the associated expansion phases in the near future. The competitive landscape revolves around multiple issues, including (1) the therapeutic target, (2) route of administration and (3) preventing/mitigating immune-mediated responses to viral vector administration. It is likely that anti-VEGF-directed gene therapies will be the first to be approved, whereas the route of administration remains more uncertain, with intravitreal, subretinal and suprachoroidal all being actively investigated and all having relative merits and disadvantages. With respect to inflammation, the nAMD patient population is significantly older than the IRD population receiving gene therapy, which may pose difficulties with tolerability to any systemic immunosuppressive drugs used in the preoperative and postoperative period, but alternatively older patients may be less likely to mount as robust an immune reaction than younger subjects.

Gene therapy allows the exciting possibility of expressing more than one active agent over time, with the capability of additional targets to VEGF, potentially resulting in greater efficacy, more sustained effect and being beneficial in patients who currently are poor responders to serial intravitreal anti-VEGFs in routine clinic use. Moreover, a greater understanding of the underlying drivers of inflammation in ocular gene therapy and its prevention/management is needed, as are developments in viral vectors that can be safely delivered intravitreally and have robust expression profiles. These two developments will arguably be game-changing, resulting in safe, readily delivered therapy to millions of patients worldwide with nAMD and potentially other common disorders including diabetic eye disease.

It is anticipated that the number of gene therapy trials will increase dramatically over the next 5 years, with cautious optimism of approved therapies within that timeframe.
